# Metabolic Stress-Induced Activation of AMPK and Inhibition of Constitutive Phosphoproteins Controlling Smooth Muscle Contraction: Evidence for Smooth Muscle Fatigue?

**DOI:** 10.3389/fphys.2017.00681

**Published:** 2017-09-08

**Authors:** Corey A. Smith, Amy S. Miner, Robert W. Barbee, Paul H. Ratz

**Affiliations:** ^1^Department of Biochemistry and Molecular Biology, Virginia Commonwealth University Richmond, VA, United States; ^2^Departments of Emergency Medicine and Physiology, Virginia Commonwealth University Richmond, VA, United States

**Keywords:** artery, bladder, cofilin, hypoxia, ischemia, myosin light chain, rhoA, phospholamban

## Abstract

Metabolic stress diminishes smooth muscle contractile strength by a poorly defined mechanism. To test the hypothesis that metabolic stress activates a compensatory cell signaling program to reversibly downregulate contraction, arterial rings and bladder muscle strips *in vitro* were deprived of O_2_ and glucose for 30 and 60 min (“starvation”) to induce metabolic stress, and the phosphorylation status of proteins involved in regulation of contraction and metabolic stress were assessed in tissues under basal and stimulated conditions. A 15–30 min recovery period (O_2_ and glucose repletion) tested whether changes induced by starvation were reversible. Starvation decreased basal phosphorylation of myosin regulatory light chain (MLC-pS19) and of the rho kinase (ROCK) downstream substrates cofilin (cofilin-pS3) and myosin phosphatase targeting subunit MYPT1 (MYPT1-pT696 and MYPT1-pT853), and abolished the ability of contractile stimuli to cause a strong, sustained contraction. Starvation increased basal phosphorylation of AMPK (AMPK-pT172) and 3 downstream AMPK substrates, acetyl-CoA carboxylase (ACC-pS79), rhoA (rhoA-pS188), and phospholamban (PLB-pS16). Increases in rhoA-pS188 and PLB-pS16 would be expected to inhibit contraction. Recovery restored basal AMPK-pT172 and MLC-pS19 to control levels, and restored contraction. In AMPKα_2_ deficient mice (AMPK$\alpha_{2}^{-/-}$), the basal level of AMPK-pT172 was reduced by 50%, and MLC-pS19 was elevated by 50%, but AMPK$\alpha_{2}^{-/-}$ did not prevent starvation-induced contraction inhibition nor enhance recovery from starvation. These results indicate that constitutive AMPK activity participates in constitutive regulation of contractile proteins, and suggest that AMPK activation is necessary, but may not be sufficient, to cause smooth muscle contraction inhibition during metabolic stress.

## Introduction

Skeletal muscle fatigue is a term used to describe the phenomenon of progressive decline in skeletal muscle force during intensive contractile activity, followed by recovery after a period of rest (Kent-Braun et al., 2012). Although fatigue has been studied for well over a century, an understanding of the precise molecular mechanisms causing fatigue, from changes in neuronal output to alterations in signaling events and contractile protein regulation, remains an area of intensive investigation. However, what is clear from these studies is that fatigue preserves cell integrity by reducing ATP hydrolysis so that cells adapt and recover rather than perish from the metabolic stress imposed by intensive activity. The cell sensor of metabolic stress, AMPK (Hardie, 2014), is activated in skeletal muscle during exercise, and AMPK participates in enhancing exercise endurance by supporting ATP synthesis and depressing ATP utilization (Lantier et al., 2014).

By comparison, smooth muscle fatigue is understudied and poorly understood. This is likely because smooth muscle contraction consumes far less ATP than even slow skeletal muscle (Walker et al., 1994), and thus, smooth muscle is generally considered fatigue-resistant. However, there are numerous examples of reversible declines in contractile force during sustained or repetitive periods of smooth muscle stimulation (Stewart, 1900; Furchgott, 1955; Ratz, 1995). Moreover, smooth muscle is dependent on an immediate supply of ATP because, unlike striated muscles that maintain high levels of phosphocreatine as an energy reserve to resupply ATP, the levels of phosphocreatine and ATP in smooth muscle are approximately equal (Ishida et al., 1994). Thus, metabolic stress, such as occurs during ischemia, hypoxia, and hypoglycemia, has the potential to compromise the ATP supply for smooth muscle contraction and, especially, for maintaining Na^+^, K^+^, and Ca^2+^ ion gradients far from their equilibrium values to prevent cell death. AMPK is activated rapidly by metabolic stress in smooth muscle (Rubin et al., 2005; Pyla et al., 2015), and activation is associated with a decline in contractile activity (Rubin et al., 2005; Goirand et al., 2007; Pyla et al., 2015).

Extreme metabolic stress induced by hemorrhage, cardiac failure, and severe sepsis lead to vasodilatory shock characterized by a “failure” of vascular smooth muscle to contract (Landry and Oliver, 2001) and involving membrane hyperpolarization (Chen et al., 2000) and desensitization of post-receptor targets (Ratz et al., 2016; Yang et al., 2017). Peripheral artery disease leading to bladder ischemia/hypoxia is proposed to play a key role in lower urinary tract syndromes such as, detrusor overactivity during filling that can lead to weak bladder smooth muscle contraction, impairing bladder voiding (Nomiya et al., 2014; Yamaguchi et al., 2014). Whether the failure of smooth muscle to contract with sufficient strength represents a form of smooth muscle fatigue remains to be determined. The present study is a first step to investigate this issue. Specifically, this study aims to determine whether AMPK activation correlates with a decline in the basal activities of the primary regulatory proteins involved in actomyosin-based contraction and force-transmission, and whether this effect is fully reversible. In short, this study is designed to determine whether smooth muscle *in vitro* can undergo fatigue (reversible decline in contraction), and whether the decline in force during metabolic stress, and recovery of force during reversal of metabolic stress, correlates with, respectively, increases and decreases in smooth muscle AMPK-pT172.

## Methods

### Animals

All studies were approved by the Institutional Animal Care and Use Committee of Virginia Commonwealth University and conform to the Public Health Service Policy on Humane Care and Use of Laboratory Animals (2015) and the National Research Council “Guide for the Care and Use of Laboratory Animals” (Eighth Edition). Specific-pathogen free, male, New Zealand White rabbits were obtained from Robinson Services, Inc., and maintained in the vivarium at 19–22°C and a 12 h light, 12 h dark cycle for at least 6–7 days prior to experimentation. Animals were individually housed, provided environmental enrichment and fed a combination of pelleted high-fiber rabbit food (Harlan Teklad 2031, ~1 cup/day) and hay. An AMPK knockout (AMPK$\alpha_{2}^{-/-}$) mouse colony was established by the Virginia Commonwealth University Transgenic/Knockout Mouse Core. Wild-type pathogen free mice were obtained from Jackson Laboratories and maintained in the vivarium at 22–23°C and a 12 h light, 12 h dark cycle for at least 3–4 days prior to experimentation. Mice (average weights ~23–30 g) were normally group housed (except for aggressive male mice, which were individually housed with added enrichment) and fed Envigo Teklad 7012 Rodent Diet *ad lib*.

### Tissue preparation

Rabbits were euthanized by ketamine/xylazine/euthasol overdose as described previously with minor modifications (Ratz, 1990). Mice were euthanized by CO_2_ asphyxiation. Immediately after sacrifice, rabbit mesentery and kidney, and mouse bladder, were rapidly removed and placed in cold (2.6°C) physiological salt solution (PSS, composition in mM: 140 NaCl, 4.7 KCl, 1.2 Na_2_HPO_4_-7H_2_O, 2.0 MOPS, 0.02 Na_2_ethylenediamine tetraacetic acid to chelate heavy metals, 5.6 D-glucose, 1.6 CaCl_2_ and 1.2 MgCl_2_, made with high-purity (17 MΩ) deionized water and adjusted using NaOH to a pH of 7.4). The renal artery (RA) and segments of mesenteric artery (MA) from the rabbit were cleaned by microdissection (Olympus SZX12) and cut into rings ~2.5 mm wide. Some rings of MA were secured in a tissue myograph (Model 610 M, Danish Myo Technology) and adjusted to the optimum length for muscle contraction (L_0_) using an abbreviated length-tension protocol in which tissues were contracted for ~5 min with a maximum concentration of KCl (110 mM KCl substituted isosmotically for NaCl) (Ratz, 1990). Bladder rings were prepared by cutting away a small section of the dome and trigone. Active contractions were normalized to the maximum active force produced at L_0_ by KCl (F/F_0_), and the force-time integral was measured by taking the area under the force-time curve.

### Phosphoprotein analysis

**Figure 1:**
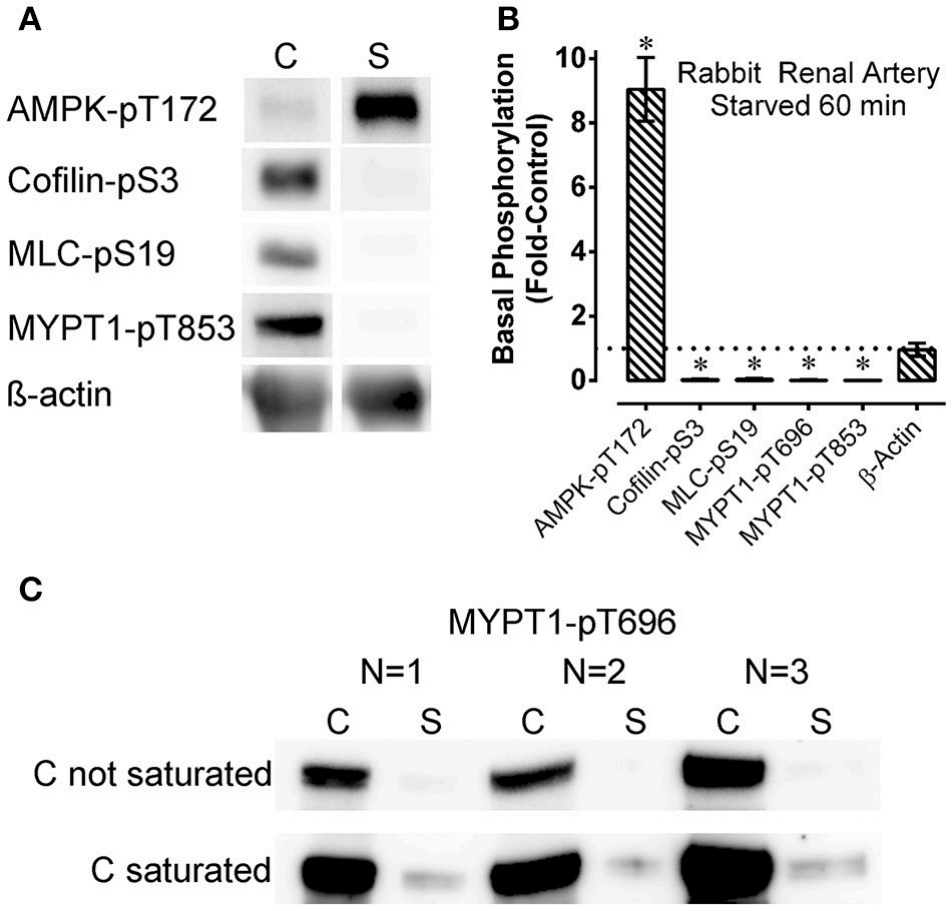
Representative Western blots **(A)**, Western blots from all 3 arteries examined **(C)**, and summary data **(B)** showing the effect of starvation (60 min of glucose and O_2_ deprivation) (“S”) compared to control (“C”) on basal levels of phosphoproteins involved in contraction regulation (cofilin-pS3, MLC-pS19, MYPT1-pT696, and MYPT1-pT853) and metabolic stress (AMPK-pT172) in renal artery. The data in **(B)** are means ± SE, *n* = 4 (*n* = 3 for MYPT1-pT696 data), of the responses to starvation (columns “S” in **A,C**) normalized to (divided by) the control responses (columns “C” in **A,C**). Upon normalization the control values are equal to 1 and the resulting starvation data are reported as “Fold-Control” (see “Methods”). ^*^P < 0.05 compared to the control value of 1. ß-actin **(A,B**) was used to assess potential variability in gel and blot quality. For MYPT1-pT696, the control bands were not saturated (“C not saturated,” **C**) and saturated (“C saturated,” **C**) to reveal the very low level of MYPT1-pT696 in the starved (“S”) tissues.

The ability of starvation to activate AMPK, and to alter phosphorylation of the downstream effectors acetyl-CoA carboxylase (ACC), myosin regulatory light chain (MLC), rhoA, cofilin, myosin phosphatase regulatory subunit (MYPT1), and phospholamban (PLB), was assessed by 1-dimensional sodium dodecylsulfate polyacrylaminde gel electrophoresis (1D SDS-PAGE) followed by “Western” blotting, enhanced chemiluminescence (ECL) and image analysis, as described previously (Ratz, 2001) with minor modifications. In all cases, for each tissue that underwent a perturbation there was a control tissue incubated for the same duration that did not undergo that perturbation and homogenates from both tissues were loaded onto the same gel. In most cases, for each gel the basal phosphorylation value (reported in arbitrary units) induced by tissue starvation was normalized to (divided by) the basal control phosphorylation value (reported in arbitrary units), and the resulting summary data were presented as fold-control. For example, for each of the four phosphoproteins and for β-actin shown in the representative Western blots in Figure 1A, the response produced by starvation (Figure 1A, column “S”) was divided by the control response (Figure 1A, column “C”). Thus, for each of the 5 rows in Figure 1A, upon normalization, each control value became “1” and the resulting summary starvation values were reported as “Fold-Control” (Figure 1B). In this way, each starvation value was statistically compared to its own control value, which was “1,” using a One-sample *t*-test (see below). In three cases where the level of basal phosphorylation was very low and near the threshold for analysis, the change in phosphorylation was normalized to a darker band produced when tissues were stimulated with phenylephrine (PE). In this case, values were reported as “Fold-PE” (see **Figures 3B, 5B,D**). In **Figure 6**, values obtained from tissues removed from the AMPK$\alpha_{2}^{-/-}$ (KO) mice (e.g., **Figure 6A**, KO column of representative Western blots) were normalized to (divided by) values obtained from tissues removed from the wild type (WT) mice (e.g., **Figure 6A**, WT column of representative Western blots), and resulting summary values were expressed as “Fold-WT.” At the appropriate time point, tissues were rapidly frozen in dry ice-cooled acetone containing 6% trichloroacetic acid, 10 mM dithiothreitol and 30 mM NaF, slowly thawed, dried, weighed and homogenized with a buffer containing 25 mM Tris- Base, 20 mM dithiothreitol, 10% glycerol, 1% sodium dodecylsulfate, 5 mM EGTA, 1 mM EDTA, 50 mM NaF, 1 mM activated Na^+^ orthovanandate, 10 μg/ml leupeptin, 10 μg/ml aprotonin and 1 mM AEBSF. Protein samples (15–20 μg/μl) were resolved by 1D SDS-PAGE and Western-blotted onto polyvinylidene difluoride membranes. The membranes were blocked with 5% bovine serum albumin at room temperature for 1 h, then incubated with an anti-phospho AMPK (pT172) antibody (1:1,000, Cell Signaling Technology, USA), anti-phospho ACC (pS79) antibody (1:1,000, Cell Signaling Technology, USA), anti-phosphocofilin (pS3) antibody (1:500, Cell Signaling Technology, USA), anti-phospho MLC (pS19) antibody (1:1,000, Sigma-Aldrich, USA), anti-phospho MYPT1 (T696 & T853) and antibodies (1:1,000, EMD Millipore, USA), anti-phospho PLB (pS16) antibody (1:1,000, Badrilla, UK), and anti-phospho rhoA (pS188) antibody (1:500, Santa Cruz Biotechnology, USA) at 4°C overnight. Goat-α-rabbit IgG- horseradish peroxidase was used as secondary antibody (1:2,000, Santa Cruz Biotechnology, USA) at room temperature for 1 h. All lanes were loaded with identical quantities of protein. However, two proteins expressed by housekeeping genes, β-actin and GAPDH, were routinely monitored to assess uniform protein loading across lanes. Data that did not display the expected equivalent protein loading were re-run or discarded. For this analysis, blots were incubated with β-actin antibody (C4) horseradish peroxidase (1:100,000, Santa Cruz, USA) and GADPH antibody (1:5,000, Santa Cruz, USA) at room temperature for 1 h. These proteins and the selected phospho-proteins were visualized using ECL (Pierce, Thermo Fisher Scientific Inc., Rockford, IL, USA) and band intensities were detected, quantified and processed using the ChemiDoc Imaging System (BioRad, Hercules, CA, USA).

### G-LISA RhoA activation assay kit for expression analysis of active RhoA (GTP bound RhoA)

Active RhoA was determined using a RhoA G-LISA™ assay as recommended by the manufacturer (Cytoskeleton Inc., Denver, CO, USA) and as previously described (Alvarez et al., 2010). Briefly, lysates prepared by homogenizing quick-frozen tissues in ice-cold lysis buffer provided by the kit were clarified by centrifugation at 25,000 × g for 10 min at 4°C, adjusted to a final concentration of 1 mg/ml protein, and an equal volume of the samples was added to the wells of the Rho G-LISA plate coated with Rho-GTP-binding protein. The plate was shaken at 400 rpm at 4°C for 30 min. After three washes at room temperature using a wash buffer supplied by the manufacturer, 50 μl of anti-RhoA primary antibody (diluted 1:250) was added to each well and left on the shaker for 45 min. The plate was washed three times, and 50 μl of diluted HRP-labeled secondary antibody (1:250) was added to the wells and placed on the shaker at room temperature for 45 min. After three washes, 50 μl of horse radish peroxidase detection reagent was added to the wells, and the luminescence signal was detected using a microplate luminescence reader (Perkin Elmer 2030 Multilabel Reader, PerkinElmer Life and Analytical Science, Turku, Finland).

### Data analysis and statistics

All data were analyzed using Graph Pad Prism 6.0 software (GraphPad Software, Inc., La Jolla, CA, USA) and are presented as representative Western blots and force tracings and as the mean ± standard error of the mean (SE). In most experiments, the data from each tissue that underwent a perturbation was normalized to the response produced by a control tissue or to a tissue that was activated by phenylephrine (PE). Western blot data from each tissue obtained from an AMPK$\alpha_{2}^{-/-}$ (KO) mouse was normalized to a corresponding tissue obtained from a wild type (WT) mouse. That is, the data were reported as a ratio in which the denominator was the value “1.” Thus, these data were evaluated by the One-sample *t*-test using a comparison to the value “1,” and the null hypothesis was rejected at *P* < 0.05. When comparing a group again, but rather than to “1,” to a second group (**Figures 3B, 5C,D**), the Student's *t*-test with the Bonferroni correction was used, so the null hypothesis was rejected at *P* < 0.025 rather than *P* < 0.05 (Glantz, 1981). In **Figure 4**, contractions induced by 10 μM PE during starvation and recovery periods were compared to control contractions (respectively, “Control-1” and “Control-2”), and evaluated by the Student's *t*-test with rejection of the null hypothesis at *P* < 0.05.

## Results

### Starvation of renal artery for 60 min

Rings of rabbit renal artery were starved for 60 min by placing them in glucose-free PSS bubbled vigorously with 100% N_2_, or retained for 60 min in an aerated, glucose-containing solution (control PSS) and quick-frozen to measure the basal levels of phosphorylation of certain contractile protein regulatory proteins, and of the metabolic stress-sensor, AMPK. After a 60 min starvation period, the basal level of AMPK-pT172 increased by ~9-fold (Figures 1A,B), indicating the starvation strongly activating the AMPK metabolic stress signal in this tissue. Concomitant with strong AMPK activation in tissues starved for 60 min the levels of basal cofilin-pS3, MLC-pS19, MYPT1-pT696, and MYPT1-pT853 were greatly reduced and barely detectable compared to the control values (Figure 1) (Ikebe and Hartshorne, 1985; Amano et al., 1996; Miyazaki et al., 2002; Muranyi et al., 2005; Bernard, 2007; Khromov et al., 2009; Mizuno, 2013; Ratz, 2016). Western blot analysis is only semi-quantitative. To examine the degree of inhibition of basal MYPT1-pT696 by starvation, blots were exposed for a longer duration that caused saturation of the control bands (Figure 1C, “C saturated”). Even under this condition, the bands representing starved tissues (“S” in Figure 1C, “C saturated”) remained nearly undetectable. In summary, the strong increase in AMPK activation and dramatic declines in basal phosphorylation levels of contractile protein regulatory proteins cofilin, MLC and MYPT1 suggest that starvation concomitantly activated the metabolic stress sensor AMPK and inactivated the smooth muscle contractile motor system.

### Recovery for 30 min of renal artery starved for 60 min

**Figure 2:**
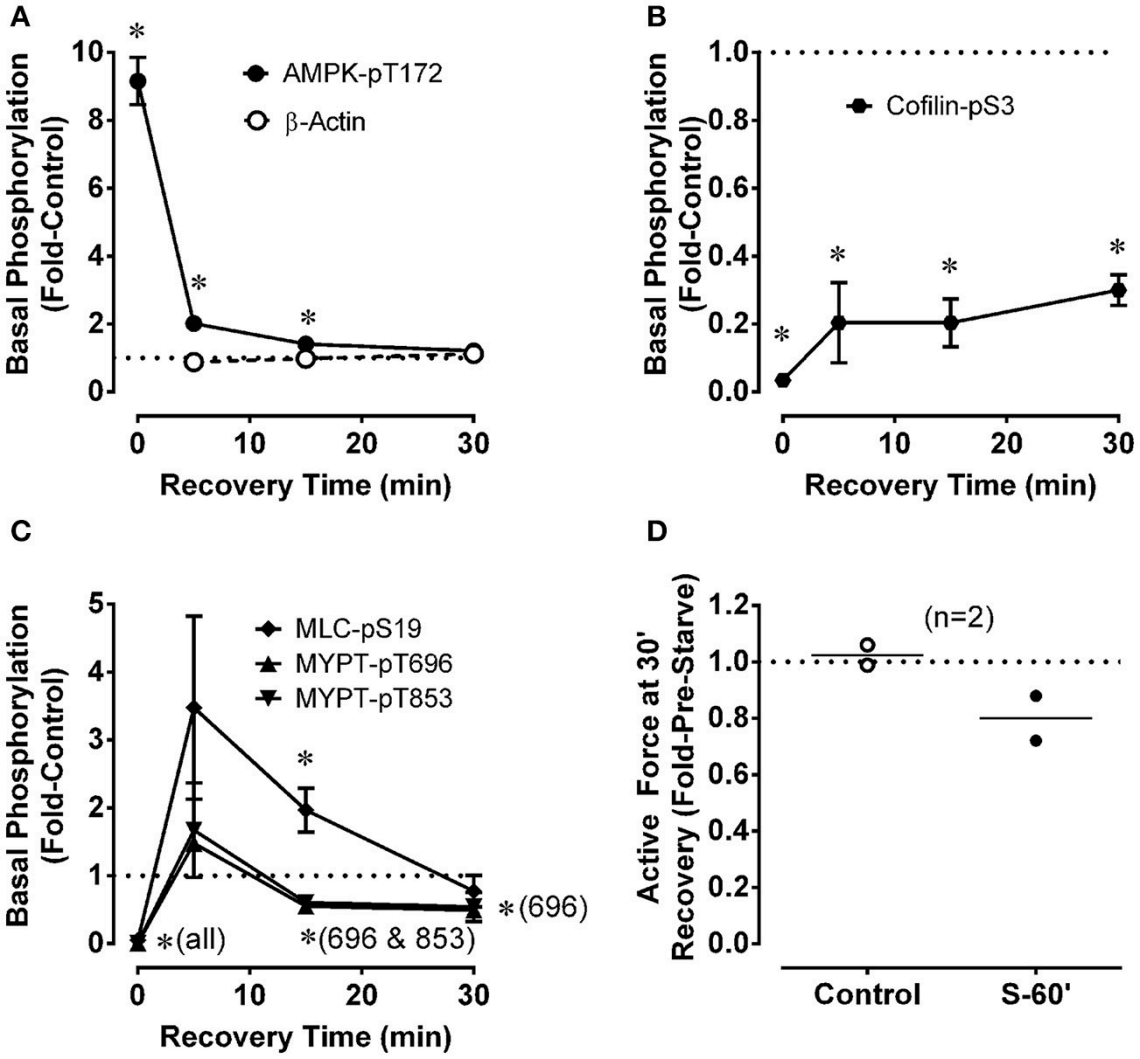
Changes for renal artery during a 30 min recovery period that followed a 60 min starvation period compared to control in the levels of phosphoproteins involved in metabolic stress (**A**, AMPK-pT172) and the regulation of actin polymerization (**B**, cofilin-pS3) and myosin activation (**C**, MLC-pS19, MYPT1-pT696, MYPT1-pT853), and in the strength of a phenylephrine-induced contraction **(D)**. ß-actin **(A)** was used to assess potential variability in gel and blot quality. Data in **(A–C)** are means ± SE, *n* = 3–4. ^*^P < 0.05 compared to the control value of 1. Panel **(D)** shows data (open and closed circles) from two rabbits along with the calculated average (horizontal lines). “S-60” refers to a 60 min starvation period.

After starvation for 60 min, rings of renal artery were placed in a recovery solution (aerated, glucose-containing, control PSS) for 5, 15, and 30 min and quick-frozen to measure indices of basal metabolic stress and basal contractile protein activation. In two tissues, the ability of renal artery to contract after 30 min of recovery was also assessed. Data from Figure 1 are reproduced as *t* = 0 data in Figures 2A–C to indicate the basal phosphorylation values after a 60 min starvation period. Basal AMPK-pT172 returned from the ~9-fold increase induced by starvation to the control basal level within 30 min (Figure 2A). Basal cofilin-pS3 increased modestly to ~25% of the control value within the first 5 min of incubation in the recovery solution, but full recovery did not occur even after 30 min (Figure 2B). Basal MLC-pS19, MYPT-1-pT696, and MYPT1-pT853 returned fully to the control level within 5 min (Figure 2C). Notably, the average level of MLC-pS19 was ~3.5-fold control at 5 min (although this apparent increase was not significantly different than control), and was significantly elevated 2-fold above control at 15 min, before returning to the control level by 30 min (Figure 2C). Following the increase to the control level within 5 min, basal MYPT1-pT696 and MYPT1-pT853 decreased to ~0.6-fold control by 15 min, and MYPT-pT696 remained at that level after the full 30 min recovery period (Figure 2C; although the average value for MYPT1-pT853 (0.54) was also < 1, this apparently low value was not significantly <1). These data reveal that, although basal AMPK-pT172 and MLC-pS19 returned rapidly and fully to the control levels during a 30 min recovery period, MLC-pS19 displayed a biphasic recovery that included an overshoot. Moreover, three other indices of contractile protein regulation, cofilin-pS3, MYPT1-pT696 and MYPT1-pT853, either did not fully recover (cofilin-pS3) or recovered within 5 min then fell to levels below the control, basal level. Despite recovery by cofilin-pS3 of only ~30%, assessment of contraction suggested that force-recovery was ~80% within 30 min (Figure 2D, *n* = 2).

### Starvation of mesenteric artery for 30 min

**Figure 3:**
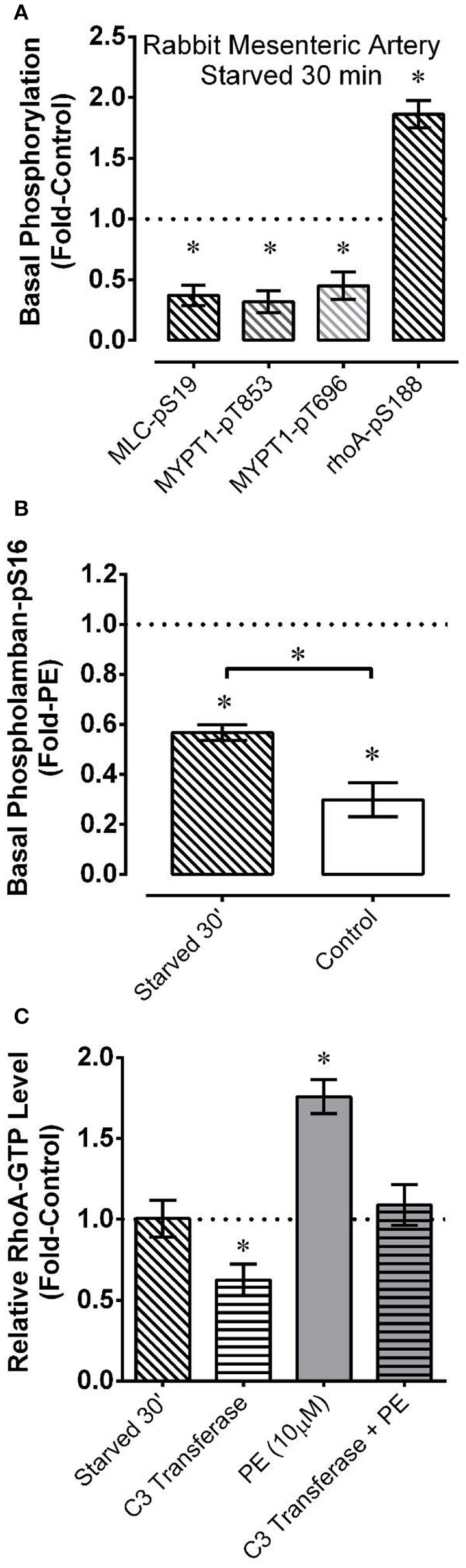
Effects measured in mesenteric artery of starvation (30 min of glucose and O_2_ deprivation) reported as “Fold-Control” or “Fold-PE” (see “Methods”) on the basal phosphorylation levels of MLC and the myosin phosphatase regulatory subunit MYPT1 **(A)**, the AMPK substrates rhoA **(A)** and PLB **(B)**, and on the level of active rhoA-GTP **(C)**. PE = phenylephrine. Data are means ± SE, *n* = 4–7 for **(A,C)**, and *n* = 3–4 for **(B)**. For **(A,C)**, ^*^P < 0.05 compared to 1. For **(B)**, ^*^P < 0.025. ^*^Over bracket indicates that bracketed groups are significantly different at *P* < 0.025.

For a comparison with renal artery, and to examine additional indices of AMPK and contractile protein activation during starvation and recovery from starvation, mesenteric artery was starved for a shorter duration (30 min) followed by recovery for 30 min. As in renal artery starved for 60 min, mesenteric artery starved for 30 min displayed strong reductions in basal MLC-pS19, MYPT1-pT696, and MYPT1-pT853 (Figure 3A), and a strong increase in AMPK-pT172 (see **Figure 5A**). Starvation for 30 min also increased the level of phosphorylation of two downstream AMPK substrates that would be expected to impact smooth muscle contractile activity, rhoA (Gayard et al., 2011; Figure 3A) and the sarcoplasmic/endoplasmic reticulum Ca^2+^-ATPase (SERCA) regulator phospholamban (PLB) (Schneider et al., 2015; Figure 3B). Notably, compared to control tissue, basal PLB-pS16 was increased by ~2-fold in starved tissue, and ~3-fold in control tissue stimulated with 10 μM phenylephrine (Figure 3B).

Alternative explanations for reductions in rhoA activity include inhibition of a guanine nucleotide exchange factor (rhoA-GEF) or activation of a GTPase activating protein (rhoA-GAP) (Wang et al., 2011). Thus, we employing a rhoA activation assay kit to determine whether a 30 min starvation period caused a reduction in the relative level of active rhoA-GTP-to-inactive rhoA-GDP. Starved mesenteric artery displayed no change in the basal rhoA-GTP/rhoA-GDP level compared to the control basal value (Figure 3C). To confirm that rabbit mesenteric artery can display changes in the relative rhoA-GTP/rhoA-GDP level, we showed that phenylephrine elevated, and C3 transferase (cell permeable ADP-ribosyltransferase) reduced the basal level of rhoA-GTP in this tissue, and that C3 transferase prevented phenylephrine from elevating the level of rhoA-GTP (Figure 3C). α-adrenergic receptor activation by phenylephrine increases rhoA-GEF activity (Lutz et al., 2005; Docherty, 2010), and C3 transferase traps rhoA in the inactive rhoA-GDP state complexed with guanine nucleotide dissociation inhibitor, preventing rhoA-GDP activation to rhoA-GTP by GEFs (Aktories et al., 2004). Thus, the absence of a change in the rhoA-GTP/rhoA-GDP levels as assessed by the rhoA assay kit supports the hypothesis that starvation did not cause a reduction in basal rhoA activity by affecting the activities of a rhoA-GEF or rhoA-GAP.

### Effect of 30 min starvation, and 30 min recovery from starvation, on contraction of mesenteric artery

**Figure 4:**
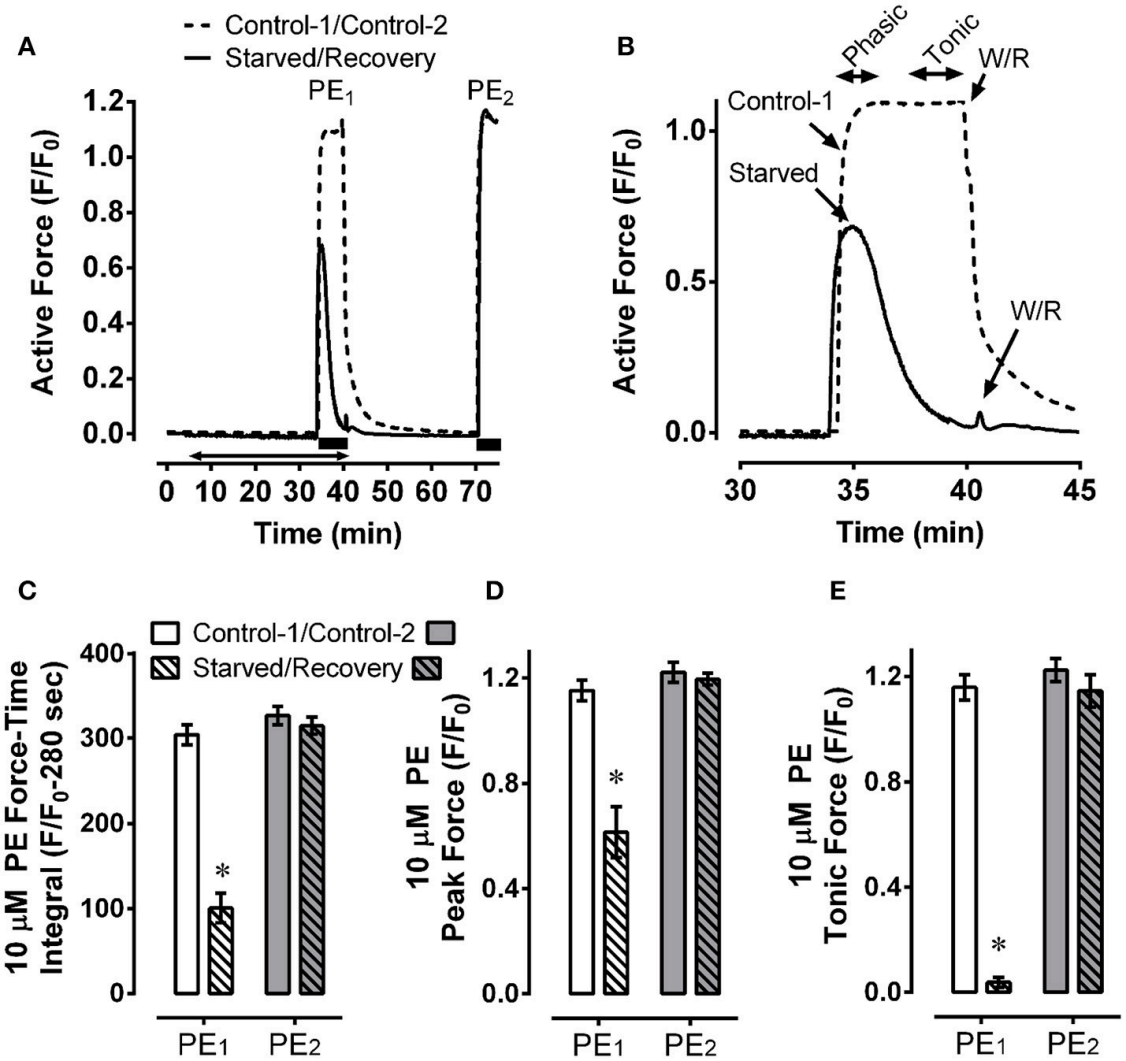
Mesenteric artery fatigue: force response. Example **(A)** and summary data **(C–E)** of two phenylephrine (PE) contractions, PE_1_ (Control-1 and Starved), and PE_2_ (Control-2 and Recovery), showing that, compared to control, starvation (30 min of glucose and O_2_ deprivation) reduced overall 10 μM PE-induced contractile strength by ~66% (**C**, PE_1_), inhibited the peak contraction by ~50% (**D**, PE_1_) and nearly abolished steady-state force maintenance (**E**, PE_1_), and that recovery from starvation (glucose and O_2_ repletion) restored early peak and steady-state force values to control levels (**A,C–E**, PE_2_). Data in **(B)** are a zoomed image of PE_1_. Data in **(C–E)** are means ± SE, *n* = 5, ^*^P < 0.05 compared to control-1. **(A)** Solid bars, duration of PE; double arrow, starvation period.

The low basal levels of MLC-pS19, MYPT1-pT696, and MYPT1-pT853 induced in starved tissues suggested to us that a contractile stimulus may be unable to produce a strong contraction in these tissues. To test this hypothesis, mesenteric artery was starved for 30 min and stimulated with 10 μM phenylephrine during the final 5 min of this period. Control tissues were treated identically but were not starved. Compared to the strong and sustained contraction produced by control tissues (Figure 4A and zoomed image, Figure 4B, dotted lines), starved tissues produced a weaker and highly transient contraction (Figure 4A and zoomed image, Figure 4B, solid lines). The resulting area under the contraction curve (force-time integral) in starved tissues was reduced by ~66% compared to control (Figure 4C, PE_1_). The early, phasic portion of contraction was reduced by ~50% (Figure 4D, PE_1_), and the sustained, tonic portion was abolished (Figure 4E, PE_1_). A subsequent 30 min recovery period induced by two washes in a normal glucose-containing, aerated, solution permitted complete recovery of contractile strength (Figures 4A,C–E, PE_2_), revealing that the inhibition of contraction induced by a 30 min starvation period was not due to irreversible tissue damage. Thus, we used the term “fatigue” for the reversible inhibition of contraction due to metabolic stress.

### Effect of 30 min starvation, and 30 min recovery from starvation, on basal and phenylephrine-stimulated phosphoprotein indices of metabolic stress and contractile protein regulation in mesenteric artery

To examine the effects of fatigue (reversible inhibition of contraction) on AMPK-pT172 and indices of contractile protein activation in mesenteric artery, tissues were treated as described in the experiment shown in Figure 4, and the levels of basal and phenylephrine-stimulated AMPK-p172, ACC-pS79 (the canonical AMPK substrate, Goransson et al., 2007), cofilin-pS3, and MLC-pS19 were assessed.

**Figure 5:**
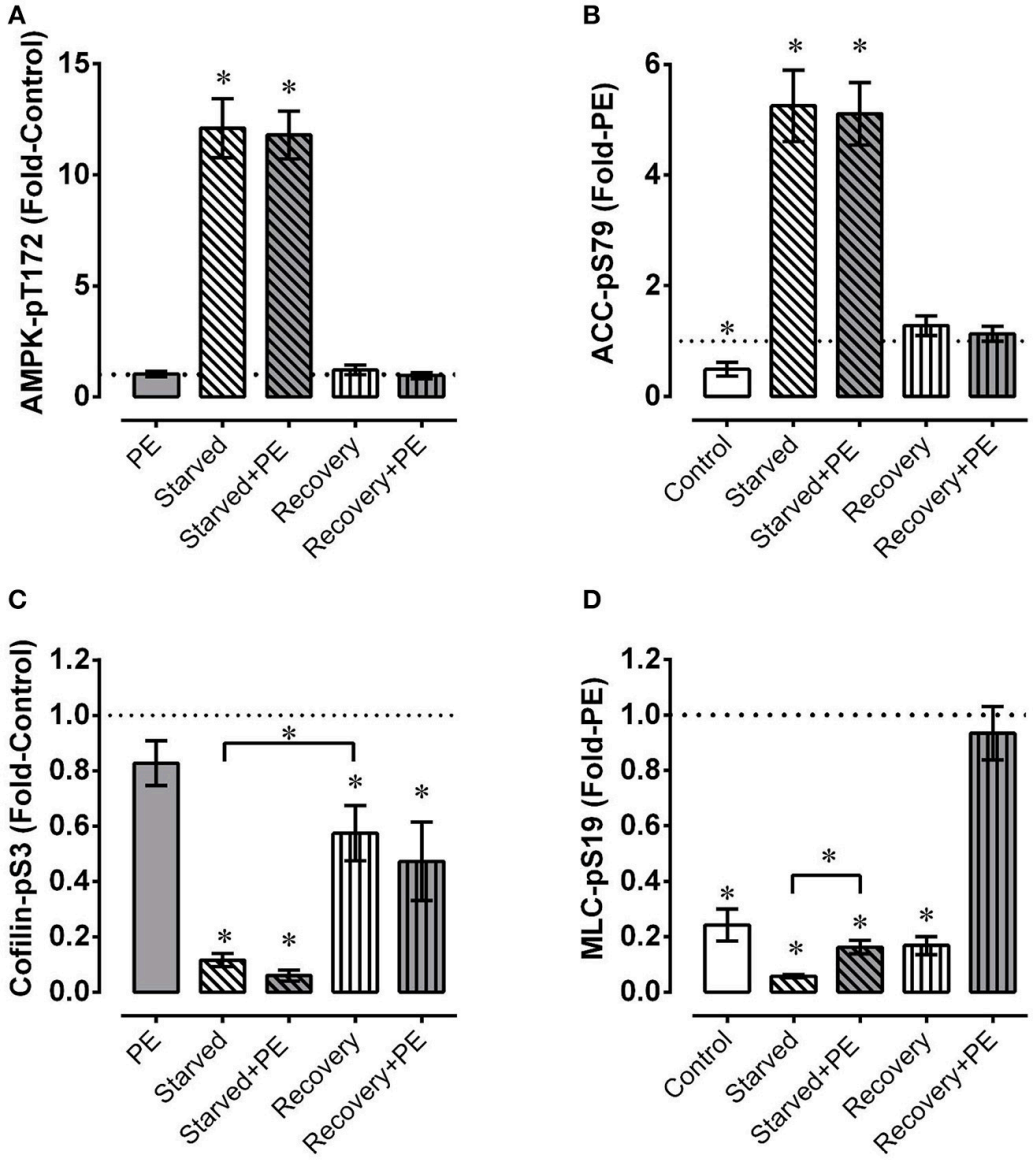
Mesenteric artery fatigue: phosphoproteins. Under basal conditions and during phenylephrine (PE) stimulation of mesenteric artery, starvation (30 min of glucose and O_2_ deprivation) elevated indices of AMPK activation (**A**, AMPK-pT172; **B**, ACC-pS79) and reduced levels of cofilin-pS3 **(B)** and MLC-pS19 **(D)**, and recovery from starvation (glucose and O_2_ repletion) largely or completely reversed these effects. Data are means ± SE, *n* = 4. ^*^P < 0.05 (and *P* < 0.025 for groups compared twice in **C,D**) compared to 1. ^*^Over bracket indicates that bracketed groups are significantly different at *P* < 0.025.

In mesenteric artery, a 5 min stimulation with phenylephrine alone did not increase AMPK-pT172 (Figure 5A, PE) but did cause an ~2-fold increase in phosphorylation of the AMPK substrate, ACC-pS79 (Figure 5B, note that all responses in panel B were normalized to the PE response, so the PE response was = 1, and the data were reported as “Fold-PE”; see “Methods”). The large increases in indices of AMPK activation induced by starvation were not further increased by phenylephrine (Figures 5A,B). Notably, AMPK-pT172 returned to the control level within 30 min of recovery (Figure 5A, Recovery). Interestingly, ACC-pS79 returned to the phenylephrine-stimulated value during the recovery phase (Figure 5B, Recovery and Recovery+PE were = 1).

As in renal artery, the level of cofilin-pS3 was greatly reduced in mesenteric artery by starvation, and recovery reversed the level toward, but not fully to, the control level (Figure 5C). However, the reversal in mesenteric artery starved for 30 min and recovered for 30 min was ~2-fold greater than that produced in renal artery starved for 60 min and recovered for 30 min (compare Figure 2B with Figure 5C, Recovery). Although the average values of cofilin-pS3 were reduced ~20% or more by phenylephrine in control tissues, during starvation, and upon recovery from starvation, these reductions were not significant (Figure 5C). Thus, the degree of metabolic stress had a dramatically greater effect on the level of cofilin-pS3 than did the contractile stimulus phenylephrine.

Phenylephrine produced an ~4-fold increase in MLC-pS19 under the control condition (Figure 5D). Interestingly, phenylephrine produced a nearly equivalent ~3-fold increase in MLC-pS19 in starved tissue. However, because the basal level of MLC-pS19 was depressed by ~80% in starved tissues, the ~3-fold increase induced by phenylephrine in starved tissues did not cause an elevation in MLC-pS19 above the control basal level. During recovery, the basal level of MLC-pS19 returned to the control basal level, and phenylephrine stimulation induced an increase that was equal to the increase induced in control tissues.

### Effect of starvation and recovery, and of AMPKα_2_ knockout, on indices of metabolic stress and contractile protein regulation of mouse bladder

**Figure 6:**
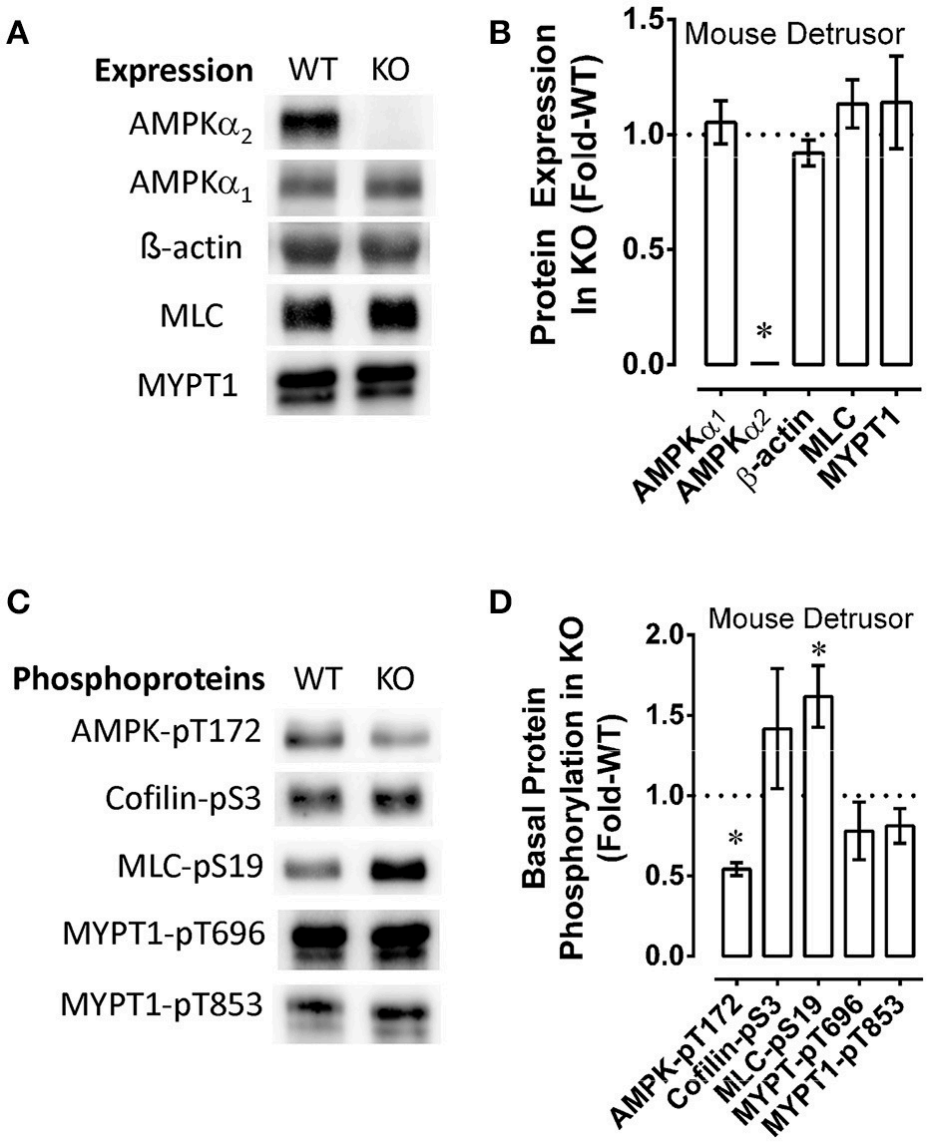
Examples **(A,C)** and summary data **(B,D)** comparing wild type (WT) and AMPK$\alpha_{2}^{-/-}$ (KO) mouse bladder protein expression **(A,B)** and basal levels of phosphoproteins **(C,D)**. KO data were normalized to (divide by) the WT data, and upon normalization, WT values are equal to 1 and the resulting “Protein Expression in KO” **(B)** and “Basal Protein Phosphorylation in KO” **(D)** are reported as “Fold-WT” (see “Methods.” Data in **B,D** are means ± SE, *n* = 4–6. ^*^P < 0.05 compared to 1.

AMPK$\alpha_{2}^{-/-}$ mouse bladder expressed the same levels of AMPKα_1_, MLC, MYPT1 and β-actin as wild-type (WT) mouse bladder and, as expected, expressed no detectable AMPKα_2_ (Figures 6A,B). Notably, compared to WT, the bladder of the AMPK$\alpha_{2}^{-/-}$ mouse displayed an 0.5-fold lower basal level of AMPK-pT172, and an 0.5-fold higher basal level of MLC-pS19 (Figures 6C,D). Basal levels of cofilin-pS3, MYPT1-pT696, and MYPT1-pT853 were not different when comparing AMPK$\alpha_{2}^{-/-}$ and WT mouse bladders (Figures 6C,D). Together, these data support the hypothesis that the basal level of MLC-pS19 is constitutively negatively regulated by the basal level of AMPK-pT172 in mouse bladder.

**Figure 7:**
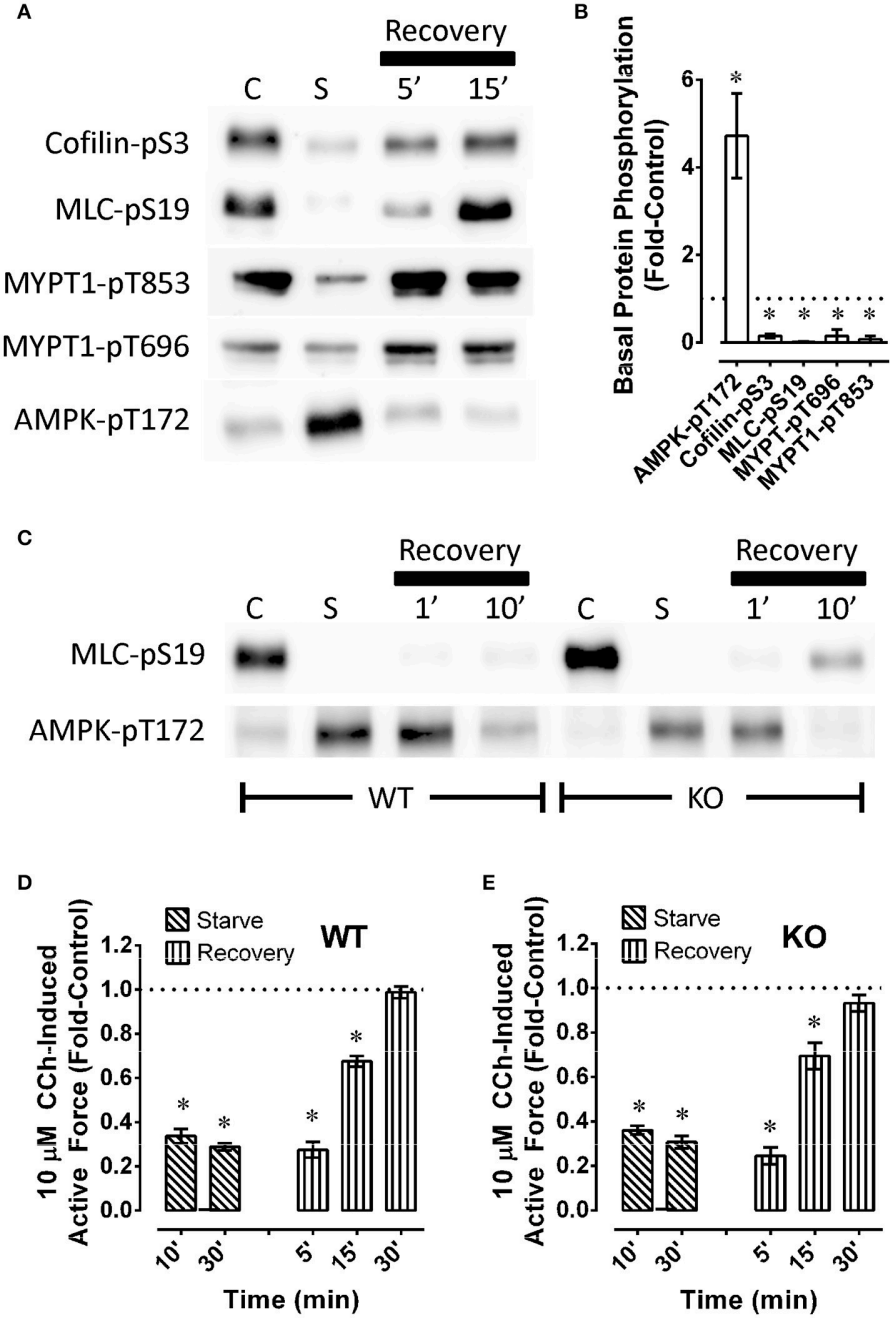
Examples **(A,C)** and summary data **(B,D,E)** of phosphoproteins **(A–C)** and carbachol (CCh)-induced maximum force **(D,E)** in wild type [**A–C** (WT) and **D**] and AMPK$\alpha_{2}^{-/-}$ [**C** (KO) and **E**] mouse bladder. In **(A,C)**, “C” and “S” refer to, respectively, control and starved, and “Recovery” refers to recovery (glucose and O_2_ repletion) from starvation (glucose and O_2_ deprivation). Data **(B,D,E)** are means ± SE, *n* = 3–6. ^*^P < 0.05 compared to 1. In **(B)**, responses to starvation (column “C” in **A**) are normalized to (divided by) control responses (column “C” in **A**) and reported as “Fold-Control” (see “Methods”).

To determine whether starvation and recovery from starvation of detrusor smooth muscle altered basal levels of phosphoproteins involved in contractile protein regulation, and the basal level of AMPK-pT172, mouse bladders were starved for 60 min or not starved (control), then permitted to recover, and quick-frozen as described above when using rabbit artery rings. As in arteries, starvation of mouse bladder increase the basal level of AMPK-pT172 and reduced basal levels of cofilin-pS3, MLC-pS19, MYPT1-pT696, and MYPT1-pT853 (Figures 7A,B). As in artery, recovery of contraction was complete within 30 min (Figure 7D), and this appeared to correlate with recovery of the basal phosphorylation states of key contractile protein regulatory proteins, and a return of AMPK-pT172 to a basal low level (Figure 7A, *n* = 1). Notably, AMPK$\alpha_{2}^{-/-}$ did not appear to prevent starvation from causing an increase in AMPK-pT172 and from abolishing basal MLC-pS19 (Figure 7C, compare WT to KO, *n* = 1), nor did it alter the degree and timing in the reduction in stimulus-induced force (compare Figures 7D,E, “Starve”) or the degree or timing in the return of contractile strength upon recovery from starvation (compare Figures 7D,E, “Recovery”).

## Discussion

The present study showed that, in smooth muscle, metabolic stress (starvation due to exposure to a glucose-free, hypoxic solution) caused a strong increase in constitutive AMPK activity concomitantly with dramatic reductions in basal levels of phosphoproteins involved in ensuring that contractile proteins are able to become activated, and reductions in the ability of stimuli to cause contraction. Notably, metabolic stress reversal by exposure of starved tissues to a glucose-containing, aerated, solution for 30 min fully returned basal levels of AMPK-pT172 and MLC-pS19 to their control values, and fully restored contraction. Thus, our data suggest that contraction can be reversibly “turned off” by metabolic stress-induced AMPK activation. These data extend those showing that starvation of rabbit bladder smooth muscle causes metabolic stress by reducing the concentration of ATP to ~60% of control within 15 min (Levin et al., 1996), that metabolic stress of smooth muscle activates AMPK and inhibits smooth muscle contraction (Rubin et al., 2005; Goirand et al., 2007; Horman et al., 2008; Gayard et al., 2011; Wang et al., 2011; Davis et al., 2012; Lee and Choi, 2013; Pyla et al., 2015; Schneider et al., 2015; Schubert et al., 2017), and that inhibition of contraction by hypoxia-induced metabolic stress is due to reductions in ROCK activity and MLC phosphorylation (Gu et al., 2005; Wardle et al., 2006, 2007). We propose that the reversible inhibition of smooth muscle contraction induced by metabolic stress that we attribute, in part, to AMPK-dependent changes in contractile protein activation may be considered a form of smooth muscle fatigue.

**Figure 8:**
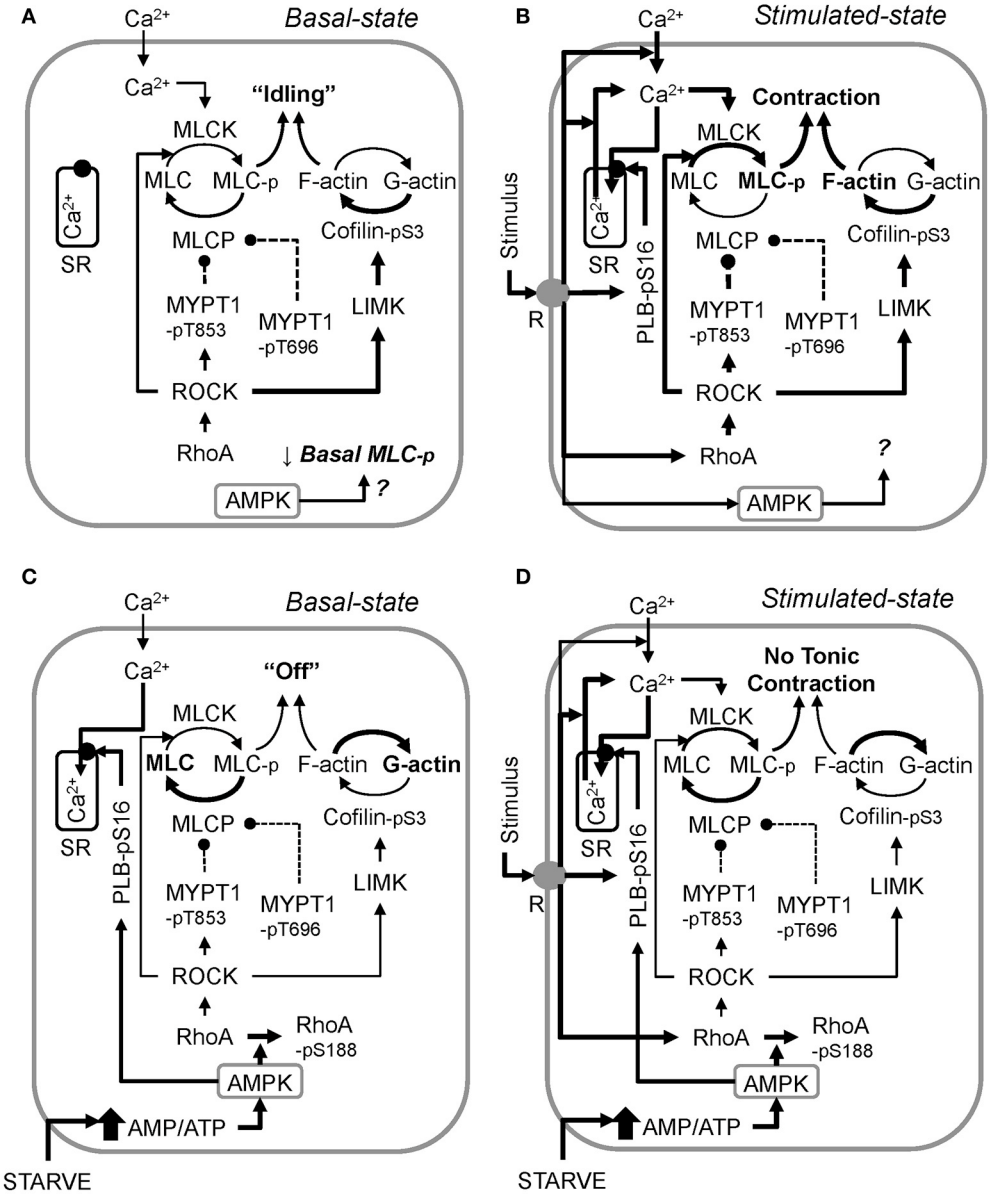
A working model of the proposed mechanism by which metabolic stress (↑ AMP/ATP ratio) “turns off” smooth muscle in the basal state (compare the control basal state, **A**, and the starved basal state, **C**), and inhibits stimulus-induced smooth muscle contraction (compare the control stimulated state, **B**, and the starved stimulated state, **D**). AMPK$\alpha_{2}^{-/-}$ data revealed that constitutive AMPK reduces basal MLC phosphorylation, but how this is accomplished remains to be determined **(A)**. Also, the precise role AMPK plays during muscle stimulation remains to be determined **(B)**. Activation of AMPK by starvation (glucose and O_2_ deprivation) increases basal Ca^2+^ sequestration into the sarcoplasmic reticulum (SR) by elevating PLB-pS16 levels, and increases basal rhoA-pS188 levels, reducing basal rhoA and ROCK activities, lowering basal levels of MYPT1-pT853 and cofilin-pS3 **(C)**. Basal MYPT1-pT696 is also lowered, but the mechanism remains to be determined. During starvation, stimuli increase MLC-pS19, but not above the control basal level. This effect, plus a potentially lower ratio of filamentous-to-globular actin (F-actin/G-actin) due to the low cofilin-pS3 levels, prevents stimuli from causing a strong contraction. Reversal of metabolic stress re-establishes normal basal enzyme activities, permitting strong force development upon stimulation. Arrows = activation, circle and dashed line = inhibition. Line thickness indicates strength of connection (thicker = stronger).

Using an AMPK$\alpha_{2}^{-/-}$ mouse, our data show for the first time that constitutive AMPK activity participates in regulating the basal level of MLC-pS19 (see Figure 6D), at least in bladder smooth muscle. In particular, because basal MLC-pS19 was elevated by ~50% in AMPK$\alpha_{2}^{-/-}$ mouse bladder, AMPK may normally act as a “brake” to constitutively reduce the level of basal MLC phosphorylation. This study also revealed that concomitant with activation of AMPK, starvation caused an increase in the basal phosphorylation levels of rhoA-pS188 and PLB-pS16, two AMPK substrates known to play a critical role in determining the basal level of MLC-pS19. Upon phosphorylation at S188, rhoA becomes complexed with GDI in the cytosol (Forget et al., 2002), even if rhoA is in the active GTP-bound conformation (Rolli-Derkinderen et al., 2005; Loirand et al., 2006) causing rhoA inactivation (Ellerbroek et al., 2003; Loirand et al., 2006). MYPT1-pT853 is a ROCK substrate (Muranyi et al., 2005), and ROCK is activated by rhoA (Amano et al., 2000). Thus, by inactivating rhoA and reducing net ROCK activity, the increase in basal rhoA-pS188 in starved mesenteric artery may have caused reductions in basal MYPT1 phosphorylation, increasing basal MLCP activity. PLB phosphorylation at S16 causes an increase in SERCA activity (Ishida and Paul, 2005), and increases in Ca^2+^ sequestration could lead to reductions in cytosolic free Ca^2+^. Basal Ca^2+^-calmodulin-dependent MLC kinase activity may participate in maintaining a basally high level of MLC-pS19. Thus, an increase in basal PLB-pS16 causing a reduction in basal cytosolic free Ca^2+^, together with an increase in basal MLCP activity due to reductions in basal MYPT phosphorylation, could explain the observed reduction in basal MLC-pS19 in starved tissue. Together, these results support the hypotheses that in smooth muscle, (1) AMPK is constitutively active and reduces basal MLC-pS19 (Figure 8A), and (2) metabolic stress greatly increases the degree of basal AMPK activation which, in turn, down-regulates contractile protein activity such that the basal contractile state of smooth muscle switches from “idling” (Figure 8A), or ready to contract when a stimulus is present (Figure 8B), to “off” (Figure 8C) and unable to contract strongly in the presence of a stimulus (Figure 8D).

Our study revealing correlative inverse changes in basal AMPK and contractile protein phosphorylation levels supports the hypothesis that AMPK played a role in causing smooth muscle fatigue. However, the data were not definitive because the rate and extent of decline and recovery in contractile strength during the fatigue protocol were not different when comparing bladders from AMPK$\alpha_{2}^{-/-}$ and control mice (see Figures 7D,E). Although basal AMPK-pT172 levels in AMPK$\alpha_{2}^{-/-}$ mouse bladder was half-that of wild type mouse bladder, starvation increased basal AMPK-pT172 and abolished MLC-pS19. Thus, it is possible that the AMPKα_1_ expressed in AMPK$\alpha_{2}^{-/-}$ mice was sufficient to cause smooth muscle fatigue. Future studies in which both AMPK isotypes are knocked out or knocked down would provide additional insight.

Smooth muscle contraction is regulated by numerous systems that converge on actomyosin crossbridges to control the activities of thick and thin filaments (Ratz, 2016). The primary mediators of thick filament regulation are Ca^2+^-dependent MLCK and rhoA-ROCK-dependent MLCP which, together, regulate the degree of MLC phosphorylation (Somlyo and Somlyo, 2003; Figure 8A). By analogy with striated muscle, smooth muscle motor proteins are generally considered to be inactive (“off”) unless the muscle is stimulated to contract. However, there is now considerable evidence that smooth muscle not stimulated to contract is made “ready” to contract by the constitutive activities of several kinases that regulate the level of resting cytosolic Ca^2+^ and MLCP activity that, in turn, maintain a relatively high basal MLC phosphorylation level (Navedo et al., 2005; Ratz and Miner, 2009; Alvarez et al., 2010; Tsai et al., 2014). In particular, the threshold permitting contraction to occur for vascular and bladder smooth muscles is ~15% MLC phosphorylation (Rembold et al., 2004). Levels of MLC phosphorylation below this threshold value do not support contraction.

Notably, kinase inhibitors and certain relaxant agents such as, forskolin can dramatically lower and even abolish basal MLC phosphorylation, and reduce basal phosphorylation levels of the negative regulators (inhibitors) of MLCP activity, MYPT1-pT696 and MYPT1-pT853 (Porter et al., 2006; Ratz and Miner, 2009; Ratz et al., 2009; Alvarez et al., 2010). We show here that, like the general kinase inhibitor staurosporine (Alvarez et al., 2010), starvation greatly reduced basal levels of MLC-pS19, MYPT1-pT696 and MYPT1-pT853 and inhibited stimulus-induced contraction. Concomitant with an increase in basal AMPK-pT172, starvation caused an increase in the basal level of the AMPK substrate rhoA-pS188, which would be expected to inhibit the active state of rhoA (Ellerbroek et al., 2003; Schneider et al., 2015), and thus, of ROCK and LIMK, resulting in reductions in basal levels of, respectively, MYPT1-pT853 and cofilin-pS3. T853 is the preferred ROCK phosphorylation site on MYPT1 (Muranyi et al., 2005), and T696 is phosphorylated by ROCK, ILK and ZIPK (Khromov et al., 2009). Thus, it is possible that inhibition of constitutive rhoA-ROCK signaling pathway by starvation caused reductions not only in basal MYPT1-pT853, but also in basal MYPT1-p696. Whether the constitutive activities of ILK and ZIPK were also affected by starvation were not pursued in this study. Low basal levels of MYPT1-pT696 and MYPT1-pT853 together would be expected to ensure that basal MLCP activity would increase, lowering basal MLC-pS19. The higher basal levels of PLB-pS16 induced by starvation may also have contributed to the lower basal MLC-pS19 because higher PLB-pS19 levels would increase sequestration of cytosolic Ca^2+^ (Ishida and Paul, 2005; Schneider et al., 2015), potentially reducing MLCK activation. Moreover, the low levels of cofilin-pS3 induced by starvation in the present study would be expected to promote actin depolymerization (Bernard, 2007), and actin depolymerization is proposed to play a role in AMPK-dependent inhibition of contraction (Schubert et al., 2017). In short, reductions in cofilin-pS3 and MLC-pS19 would be expected to reduce the ability of smooth muscle to contract because higher levels of cofilin-pS3 enhance the formation and stability of filamentous actin (F-actin) (Mizuno, 2013), and MLC-pS19 is necessary for smooth muscle contraction (Ikebe and Hartshorne, 1985). We propose that such actions would effectively shift smooth muscle motor proteins from an “idling” state (Figure 8A) to an inactive, “off,” state (Figure 8C), and thus, would significantly attenuate the ability of stimuli to “turn on” smooth muscle motor proteins (compare Figures 8B,D).

Upon phosphorylation at S188, rhoA becomes complexed with GDI in the cytosol (Forget et al., 2002), even if rhoA is in the active GTP-bound conformation (Rolli-Derkinderen et al., 2005; Loirand et al., 2006) causing rhoA inactivation (Ellerbroek et al., 2003; Loirand et al., 2006). MYPT1-pT853 is a ROCK substrate (Muranyi et al., 2005), and ROCK is activated by rhoA (Amano et al., 2000). Thus, by inactivating rhoA and reducing net ROCK activity, the increase in basal rhoA-pS188 in starved mesenteric artery may have caused reductions in basal MYPT1 phosphorylation, increasing basal MLCP activity. PLB phosphorylation at S16 causes an increase in SERCA activity (Ishida and Paul, 2005), and increases in Ca^2+^ sequestration could lead to reductions in cytosolic free Ca^2+^. Basal Ca^2+^-calmodulin-dependent MLC kinase activity may participate in maintaining a basally high level of MLC-pS19. Thus, an increase in basal PLB-pS16 causing a reduction in basal cytosolic free Ca^2+^, together with an increase in basal MLCP activity due to reductions in basal MYPT phosphorylation, could explain the observed reduction in basal MLC-pS19 in starved tissue.

In summary, we propose that the AMPK signaling system reflects a smooth muscle compensatory mechanism to reduce energy demand and sustain cell integrity during reduced energy supply. That is, the present study supports the hypothesis that AMPK acts as a signal to induce fatigue in smooth muscle and preserve muscle function for some time while under the compromised metabolic state. Although each has a distinct initiating cause, hemorrhage, cardiac failure and sepsis can culminate in vasodilatory shock characterized by vascular hyporeactivity of unknown etiology (Bond et al., 1986; Chen et al., 2000; Landry and Oliver, 2001; Liu et al., 2003; Gomez et al., 2012; Ratz et al., 2016; Yang et al., 2017). Moreover, although the mechanism of bladder underactivity remains to be determined, bladder ischemia/hypoxia has been proposed to play a causative role. The present study provides incentive to perform additional studies to determine whether AMPK-activated smooth muscle fatigue plays a role in smooth muscle disorders such as, vasodilatory shock and bladder underactivity.

## Author contributions

CS was responsible for protocol design, implementation of experiments, data acquisition, and revising the manuscript. AM was responsible for experiments and data acquisition. RB assisted with project conceptualization, protocol development, and revising the manuscript for intellectual content. PR was responsible for project conceptualization, protocol development, data analysis and interpretation, writing and revising the manuscript, and for final approval of the completed article. All authors read and approved the final manuscript.

### Conflict of interest statement

The authors declare that the research was conducted in the absence of any commercial or financial relationships that could be construed as a potential conflict of interest.

## Abbreviations

ACC: acetyl-CoA carboxylase
AMPK: AMP-dependent protein kinase
GAP: GTPase activating protein
GEF: guanine nucleotide exchange factor
MLC: myosin regulatory light chain
MYPT1: myosin phosphatase targeting subunit 1
PE: phenylephrine
PLB: phospholamban
PSS: physiological salt solution
ROCK: rho kinase
SERCA: sarcoplasmic and endoplasmic reticulum calcium ATPase.
